# Memory CD8^+^ T cells exhibit tissue imprinting and non‐stable exposure‐dependent reactivation characteristics following blood‐stage *Plasmodium berghei* ANKA infections

**DOI:** 10.1111/imm.13405

**Published:** 2021-08-27

**Authors:** Tovah N. Shaw, Michael J. Haley, Rebecca S. Dookie, Jenna J. Godfrey, Antonn J. Cheeseman, Patrick Strangward, Leo A. H. Zeef, Ana Villegas‐Mendez, Kevin N. Couper

**Affiliations:** ^1^ Faculty of Biology, Medicine and Health The Lydia Becker Institute of Immunology and Inflammation University of Manchester Manchester UK; ^2^ School of Biological Sciences Institute of Immunology and Infection University of Edinburgh Edinburgh UK; ^3^ Faculty of Biology, Medicine and Health University of Manchester Manchester UK

**Keywords:** brain, CD8^+^ T cell, cerebral malaria, immune memory

## Abstract

Experimental cerebral malaria (ECM) is a severe complication of *Plasmodium berghei* ANKA (PbA) infection in mice, characterized by CD8^+^ T‐cell accumulation within the brain. Whilst the dynamics of CD8^+^ T‐cell activation and migration during extant primary PbA infection have been extensively researched, the fate of the parasite‐specific CD8^+^ T cells upon resolution of ECM is not understood. In this study, we show that memory OT‐I cells persist systemically within the spleen, lung and brain following recovery from ECM after primary PbA‐OVA infection. Whereas memory OT‐I cells within the spleen and lung exhibited canonical central memory (Tcm) and effector memory (Tem) phenotypes, respectively, memory OT‐I cells within the brain post‐PbA‐OVA infection displayed an enriched CD69^+^CD103^−^ profile and expressed low levels of T‐bet. OT‐I cells within the brain were excluded from short‐term intravascular antibody labelling but were targeted effectively by longer‐term systemically administered antibodies. Thus, the memory OT‐I cells were extravascular within the brain post‐ECM but were potentially not resident memory cells. Importantly, whilst memory OT‐I cells exhibited strong reactivation during secondary PbA‐OVA infection, preventing activation of new primary effector T cells, they had dampened reactivation during a fourth PbA‐OVA infection. Overall, our results demonstrate that memory CD8^+^ T cells are systemically distributed but exhibit a unique phenotype within the brain post‐ECM, and that their reactivation characteristics are shaped by infection history. Our results raise important questions regarding the role of distinct memory CD8^+^ T‐cell populations within the brain and other tissues during repeat *Plasmodium* infections.

## Graphical abstract

Antigen‐specific memory CD8^+^ T cells are sustained systemically but exhibit a distinct phenotype within the brain post‐experimental cerebral malaria. Memory parasite‐specific CD8^+^ T cells initially reactivate strongly following parasite reinfection but are quickly repressed upon repetitive reinfections.

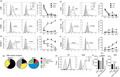

**Abbreviations**
ACT: artesunate and chloroquine antimalarial drug treatment
ECM: experimental cerebral malaria
GFP: green fluorescent protein
GO: gene ontology
GrB: Granzyme B
HCM: human cerebral malaria
ICOS: inducible T‐cell costimulator
IFN: interferon
KLRG‐1: Killer cell lectin‐like subfamily G member 1
OVA: ovalbumin
PbA: *Plasmodium berghei* ANKA
pRBC: parasitized red blood cells
T‐bet: T‐box transcription factor 21
Tcm: central memory T cell
tdTe: terminally differentiated effector T cell
Te: effector T cell
Tem: effector memory T cell
Trm: resident memory T cell

## INTRODUCTION

Human cerebral malaria (HCM) is a major cause of mortality following *Plasmodium falciparum* infection, responsible for approximately 300 000 deaths annually [1]. The *Plasmodium berghei* ANKA (PbA) model of experimental cerebral malaria (ECM) has been extensively utilized to study the pathogenesis of malaria‐induced neuropathology (reviewed [2, 3]). ECM is a multifactorial condition characterized by wide‐spread disruption to the blood–brain barrier with resultant vasogenic oedema and brain swelling, significant cerebrovascular congestion and haemostasis, cerebral haemorrhaging and parenchymal pathologies, including axonal injury [4]. The pathophysiology of ECM is complex, and the spatiotemporal sequence of events underlying its genesis remains incompletely understood; however, accumulation of parasitized red blood cells (pRBC) and leucocytes within (and around) the brain vasculature network appears to be important events in the development of the syndrome [2, 3]. Specifically, a pivotal role for CD8^+^ T cells in development and progression of the ECM syndrome has been identified [5, 6].

During primary PbA infection in ECM‐susceptible strains of mice, such as C57BL/6 mice, pathogenic parasite‐specific CD8^+^ T cells are primed in the spleen by cross‐presenting CD8^+^ Clec9a^+^ DCs [7, 8]. The activated CD8^+^ T cells then migrate to the brain in a CXCR3‐CXCL10‐dependent manner and form long‐lasting cognate interactions with parasite antigen cross‐presenting endothelial cells, with CXCL10 playing an additional pathological role in the brain by stabilizing the binding of CD8^+^ T cells with the endothelial cells [9, 10, 11, 12, 13, 14]. IFN‐γ plays an important role in ECM pathogenesis by promoting endothelial cell cross presentation, MHC‐class I expression and CXCL10 production within the brain, facilitating CD8^+^ T‐cell entry and retention in the brain [10, 11, 15, 16]. Following interaction with parasite‐cross‐presenting endothelial cells, CD8^+^ T cells mediate disruption of the blood brain barrier through Perforin and Granzyme B (GrB) production [17, 18, 19]. Notably, potential roles for CD8^+^ T cells in HCM has recently been described, with CD8^+^ T cells found in similar numbers in the brains during fatal HCM as is observed in ECM [20, 21]. Moreover, CD8^+^ T cells appear to degranulate and release GrB proximal with brain endothelial cells in HCM [21], and the proportions of CD8^+^ T cells producing GrB were significantly higher in children with severe malaria than in those with uncomplicated malaria [22].

Whilst ECM is generally a fatal condition, it can be treated, suboptimally, by the administration of antimalarial drugs [23, 24]. How the brain recovers following an episode of ECM is poorly understood. In particular, whether parasite‐specific pathogenic CD8^+^ T cells are retained or operate an enhanced immune surveillance programme within the brain post‐ECM, compared with other affected tissue sites such as the lung, has yet to be examined. Of relevance, conflicting studies have shown that systemic infections may lead to short‐term CD8^+^ T‐cell immunosurveillance or long‐term tissue‐resident memory (Trm) CD8^+^ T‐cell responses in the brain [25, 26]. Moreover, Trm CD8^+^ T cells, characterized by the expression of the c‐type lectin CD69 and the integrin CD103, have been observed to reside within the brain following various cerebral infections [27, 28, 29, 30, 31, 32]. Interestingly, brain‐resident memory CD8^+^CD103^+^ T cells exhibit a unique gene signature as compared with other memory CD8^+^ T‐cell populations within different tissue sites [27, 29], which is consistent with the model whereby intracerebral CD8^+^ T cells receive imprinting upon entry into the brain, promoting specialized functions [33].

In this study, we have directly investigated the distribution and fate of parasite‐specific CD8^+^ T cells following an episode of ECM. We show that memory CD8^+^ T cells are widely spread following drug treatment of ECM, including in the brain, and that intracerebral memory CD8^+^ T cells exhibit a distinct CD69^+ ^CD103^−^ memory phenotype compared with memory cells in the spleen and lung. Importantly, we demonstrate that memory CD8^+^ T cells reactivate very strongly and dominate the splenic and intracerebral CD8^+^ T‐cell responses during secondary challenge PbA infection, to the exclusion of new effector CD8^+^ T cells. However, we also reveal that memory CD8^+^ T‐cell reactivation and pathogenicity is significantly reduced following multiple repetitive PbA infections, indicating that memory CD8^+^ T‐cell characteristics are not stably imprinted upon parasite re‐exposure. Combined, our results demonstrate that memory CD8^+^ T cells are maintained with apparent tissue‐specific imprinting post‐PbA infection, and that the regulation of memory CD8^+^ T‐cell reactivation is a key event governing the outcome of repeat PbA infections.

## MATERIALS AND METHODS

### Ethics

All animal work was approved by the University of Manchester Animal Procedures and Ethics committee and was performed in accordance with the UK Home Office (HO) Animals (Scientific Procedures) Act 1986 (HO Project licences 70/7293 and P8829D3B4).

### Parasites and infections

C57BL/6 mice were purchased from Charles River, UK. CD45·1^+/−^ OT‐I mice and CD45·1^+/+^ OT‐I mice were generated and bred at the University of Manchester by crossing CD45·1^+^ (Pep3) mice with RAG‐1 OT‐I mice to F1 and F2 generations, respectively. Resultant mice were genotyped by assessing CD45·1/CD45·2 expression and presence of the OT‐I TCR (V_α_2 and V_β_5) by flow cytometry. Mice were maintained in individual ventilated cages at the University of Manchester.

Cryopreserved transgenic PbA parasites expressing ova epitopes (OVA_323–339_[KISQAVHAAHAEINEAG]) and GFP under control of the *P*. *berghei* elongation factor (EF)‐1a promoter (PbTg parasites referred to in this study as PbA‐OVA) [34], kindly provided by Professor Heath, University of Melbourne, were thawed and passaged once through C57BL/6 mice. Experimental animals were infected intravenously (i.v.) with 1 × 10^4^ pRBC. PbA‐OVA parasites were maintained under pyrimethamine selection *in vivo* in both passage and experimental mice, as previously described [34]. Peripheral parasite levels were assessed every 2nd day of infection by examination of Giemsa‐stained thin blood smears.

Animals were drug‐cured by six consecutive daily intraperitoneal (i.p.) injections of chloroquine and artesunate (both 30 mg/kg), commenced when animals showed prodromal signs of ECM, typically on day 6 of infection, as previously described [23, 24]. In some experiments, animals were re‐infected on day 60 post‐infection with 1 × 10^4^ PbA‐OVA pRBCs, as described above. In specific experiments, animals were injected i.p. with 250 μg anti‐CD8 mAb (clone 53‐6·72) or control rat IgG2a Ab for two consecutive days on day 60 post‐primary infection to examine the capacity of systemically administered anti‐CD8α mAb to target memory CD8^+^ T cells, or were injected on days −1, 1, 3, 5 and 7 of secondary challenge infection to examine the role of blood‐accessible memory CD8^+^ T cells in promoting ECM during challenge infection. In other experiments, animals were injected i.v. with 3 μg anti‐CD45‐FITC (30‐F11) on day 60 post‐infection, and were culled after 3 min to examine *in vivo* labelling of intravascular cells [35]. Antibodies for *in vivo* administration from BioXcell (West Lebanon, NH). To generate repeatedly infected mice, mice were treated with antimalarial drugs during primary infection, as described above, when they exhibited prodromal signs of ECM and this cycle was repeated up to three times, with a minimum of 30 days between clearance of parasites and re‐infection [23].

ECM was graded as previously described [36]: 1 = no signs; 2 = ruffled fur/and or abnormal posture; 3 = lethargy; 4 = reduced responsiveness to stimulation and/or ataxia and/or respiratory distress/hyperventilation; 5 = prostration and/or paralysis and/or convulsions. Stages 2 and 3 were classified as prodromal ECM, and stages 4–5 were classified as ECM. PbA‐infected mice were euthanized when they reached stage 4/5.

### Flow cytometry

Spleens were removed and single‐cell suspensions created by homogenizing the tissue through a 70‐µm cell sieve (BD Biosciences). Brains and lungs were isolated from mice following intracardiac perfusion with PBS. Gentle intracardiac perfusion does not remove extravascular or intravascular effector or memory T‐cell populations that are adherent to endothelial cells within either the brain or lung, and as such removes blood‐associated cells that have not been recruited to the tissues [12, 37]. Tissues were chopped into small pieces, passed through a 10‐ml syringe and incubated in HBSS containing 2% FCS with Collagenase D (final concentration 1 mg/ml; Sigma) for 45 min on a tube roller at room temperature. The resultant cell suspensions were filtered through a 70‐µm cell sieve. Brain cell suspensions were layered on a 30% Percoll gradient and centrifuged at 2000*g* for 10 min, the supernatant was discarded, and the cell pellet collected. RBCs were lysed in tissue samples by addition of RBC lysing buffer (BD Biosciences) and were washed and resuspended in HBSS containing 2% FCS (FACS buffer). Lung cell suspensions were refiltered through a 70‐μm cell sieve to remove aggregates. Absolute cell numbers were determined by microscopy using a haemocytometer, and live/dead differentiation was performed using the trypan blue exclusion cell viability assay (Sigma).

Isolated leucocytes were surface stained for 20 min with α‐mCD3 (17A2), CD8 (53‐6·7), KLRG‐1 (2F1), ICOS (15F9), CD127 (A7R34), CD103 (2E7), CD69 (H1.2F3), CD44 (IMF), CD62L (MEL‐14), CD45·1 (A20), CD45·2 (104), PD‐1 (RMP1‐30) and LAG‐3 (C9B7W). Intracellular staining for GrB (GB11) and Ki67 (SolA15) was performed for 45 min, after treatment with Foxp3 fixation/permeabilization buffer (eBioscience). Dead cells were excluded using LIVE/DEAD^®^ Fixable Blue Dead Cell Stain Kit (Life Technologies). Fluorescence minus one controls were used to set gates. Cells were analysed with a BD LSR II (Becton Dickinson) using BD FACSDiva software (Becton Dickinson). Data were analysed with FlowJo (Tree Star Inc.). All antibodies were from eBioscience, Biolegend or BD Biosciences.

### OT‐I adoptive transfer

Splenic single‐cell suspensions were prepared from CD45·1^+/+^ OT‐I mice and CD45·1^+/−^ OT‐I mice, as described above. CD8^+^ T cells (OT‐Is) were negatively selected by staining splenocytes with anti‐mCD4 (GK1·5), F4/80 (BM8), MHC‐II (M5/114·15·2) and CD19 (eBio1D3), all conjugated to phycoerythrin (PE), and using anti‐PE conjugated midiMACS beads, according to the manufacturer's instructions (Miltenyi Biotec). The flow through containing CD8^+^ T cells was collected and was examined by flow cytometry (cells 95% T cell receptor αβ^+^ and predominantly CD44^−^CD62L^+^). In the majority of experiments, 10 000 CD45·1^+/−^ OT‐I cells were adoptively transferred into naïve C57BL/6 mice 1 day prior to primary infection with PbA‐OVA. In some experiments, 10 000 CD45·1^+/+^ OT‐I cells were adoptively transferred to C57BL/6 mice on approximately day 60 post‐PbA‐OVA infection prior to secondary re‐infection.

### Immunohistochemistry

Brains were sectioned at 10 µm on a cryostat (Leica), mounted on SuperFrost Plus slides (Thermo Fisher) and fixed with 4% paraformaldehyde for 10 min. Slides were washed with PBS, and non‐specific binding blocked (1% BSA, 0·05% Tween‐20 in PBS) for 1 h, followed by overnight incubation at 4 °C with Alexa Fluor^®^ 647 anti‐mouse CD45·1 (1:100, BioLegend) and PE anti‐mouse CD31 (1:100, BioLegend). Slides were then washed with PBS, incubated with DAPI (0·5 µg/ml) for 30 min, washed again with PBS and covered‐slipped with ProLong Gold (Invitrogen). Images were captured using an Olympus BX63 upright microscope through CellSens Dimension v1·16 (Olympus).

### RNA‐seq and bioinformatics

The whole‐brain RNA‐seq database of PbA chemotherapy utilized in this study and the mRNA quality control, sequencing, mapping and the bioinformatics analysis have previously been described [24], Array Express accession no. E‐MTAB‐6474. Briefly, genes of interest were identified by pairwise comparisons between d0 samples and all other groups (< or >1·5 fold change and q value ≤0·05). For time course visualization, gene expression in each group was normalized to the d0 average. Gene ontology (GO) enrichment analysis on the differentially expressed gene lists was performed using The Gene Ontology Resource (powered by Panther). Gene network analysis on the differentially expressed gene lists was performed using IPA.

### Statistical analysis

The distribution of the data was analysed using the Shapiro–Wilk test. For two group comparisons, statistical significance was determined using an unpaired two‐tailed *t* test (for parametric data) or the Mann–Whitney *U* test (for non‐parametric data). For three or more group comparisons, statistical significance was determined using a one‐way ANOVA, with Tukey post hoc analysis, or a Kruskal–Wallis test, with Dunn post hoc analysis, for parametric and non‐parametric data, respectively. Results were considered as significantly different when *p* < 0·05.

## RESULTS

### Parasite‐specific CD8^+^ T cells persist in different tissues including the brain post‐ECM

**FIGURE 1:**
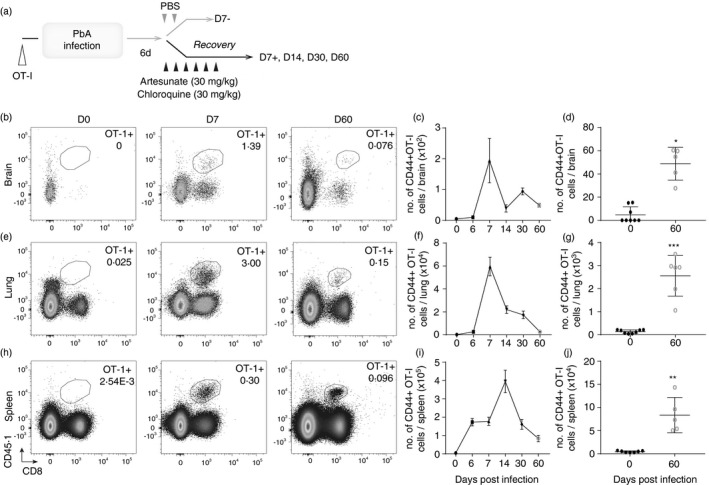
Antigen‐specific CD8^+^ T cells persist in various tissues, including the brain, post‐ECM. 10 000 naïve CD45·1^+^OT‐I cells were adoptively transferred (i.v.) into CD45·2^+^C57BL/6 mice 1 day prior to infection with 10^4^ PbA‐OVA pRBCs. The mice were treated (i.p.) with antimalarial drugs (artesunate (30 mg/kg) and chloroquine (30 mg/kg)) when they exhibited signs of ECM. (a) Schematic of the experiment. Brain, lungs and the spleen were removed on various days preceding and post‐PbA‐OVA infection. (b, e, h) Representative histograms showing the identification of CD45·1^+^ OT‐I cells in the different organs. (c, f, i) The numbers of CD44^+^OT‐I cells in the tissues over the course of the experiment and (d, g, j) specifically on day 60 post‐infection compared with naïve mice. Results are from one experiment of three independent experiments. Results are the mean ± SEM of the group (with *n* = 5–8 per group). (d, g, j) Unpaired *t* test. *p < 0·05, **p < 0·001, ***p < 0·0001

CD8^+^ T‐cell migration to the brain is a signature of ECM [5, 6] but whether CD8^+^ T cells persist within the brain long term and at a comparable level to other tissue sites following antimalarial drug treatment (ACT) is unknown. To investigate this, we adoptively transferred OT‐I cells into mice prior to infection with PbA‐OVA parasites and administered combined artesunate and chloroquine ACT on day 6 post‐infection, when mice showed signs of prodromal ECM (experimental schematic shown in Figure 1a). Antimalarial drug‐treated mice developed significant cerebral pathology, including blood–brain barrier breakdown, haemorrhage and axonal injury (as shown in [24]); however, the majority (80%) of drug‐treated mice rapidly recovered by day 8 of infection and cleared patent peripheral parasites by day 10 (as shown in [24]).

As expected, the numbers of activated OT‐I CD44^+^ cells (representative dot plots showing identification in Figure 1b and gating strategy in Figure S1A) peaked in the brain immediately post‐drug treatment on day 7 of infection (Figure 1c). The numbers of intracerebral OT‐I CD44^+^ cells in the brains of drug‐treated mice on day 7 of infection were significantly lower than in non‐drug‐treated mice experiencing fatal ECM, whereas the number of splenic OT‐I CD44^+^ cells on day 7 of infection was not significantly affected by drug treatment (Figure S1B). Therefore, antimalarial drug administration reduced but did not completely abrogate OT‐I CD44^+^ cell migration to and accumulation in the brain. The numbers of intracerebral OT‐I CD44^+^ cells subsequently declined rapidly from the day 7 peak of response (Figure 1c); although significantly increased numbers of OT‐I cells were maintained within the brain on day 60 p.i. (compared with numbers in naïve mice), numbers were very low (Figure 1d). A similar trend in OT‐I CD44^+^ expansion and extreme contraction was observed in the lung (Figure 1e‐g), a comparator non‐lymphoid organ that experiences T cell‐mediated immunopathology during *P*. *berghei* infection [38, 39]. The OT‐I CD44^+^ cell response peaked later on day 14 post‐infection in the spleen, the major site of T‐cell priming and the dominant organ for effector T‐cell responses during malaria [40], compared with the lung and brain and, as expected, a large OT‐I CD44^+^ cell population was maintained in the spleen on day 60 pi (Figure 1h–j). Overall, these data show that memory OT‐I CD44^+^ cells are retained in the brain as well as the lung and spleen long‐term post‐ECM.

### Parasite‐specific CD8^+^ T cells exhibit a distinct memory phenotype in the brain post‐ECM

Memory T‐cell responses are frequently heterogeneous, displaying varying phenotypic and functional characteristics within differing tissues, which can relate to T‐cell adaptation to local environmental conditions and cellular migratory (circulatory vs. tissue‐resident) patterns [41, 42]. For example, memory T cells in the brain can have distinct phenotypic and functional profiles compared with memory T cells in other tissues [27, 29]. Consequently, we investigated whether the memory OT‐I cells retained in the brain post‐PbA‐OVA infection were comparable or different from the memory OT‐I cells in the spleen and lung.

**FIGURE 2:**
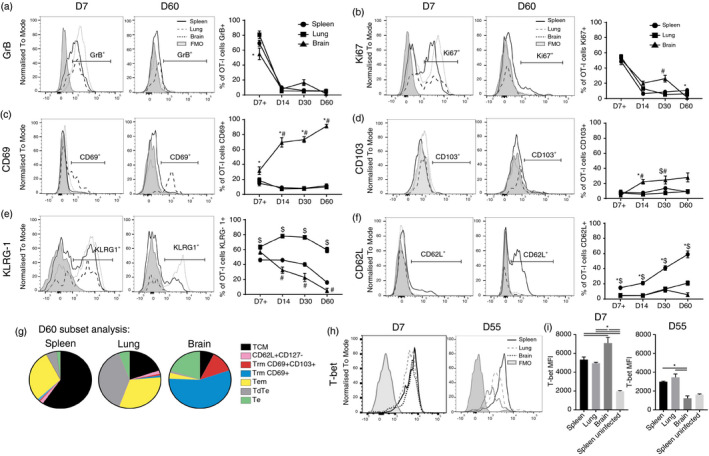
Memory OT‐I cells exhibit tissue‐specific signatures post‐ECM. 10 000 naïve CD45·1^+^OT‐I cells were adoptively transferred (i.v.) into CD45·2^+^C57BL/6 mice 1 day prior to infection with 10^4^ PbA‐OVA pRBCs. The mice were treated (i.p.) with antimalarial drugs (artesunate (30 mg/kg) and chloroquine (30 mg/kg)) when they exhibited signs of ECM. Representative histograms and proportions of CD44^+^OT‐I cells expressing (a) GrB, (b) Ki67, (c) CD69, (d) CD103, (e) KLRG‐1 and (f) CD62L within the spleen, lung and brain post‐ECM (FMO from spleen). (g) The subdivision of the CD44^+^OT‐I cell populations into canonical effector/memory T cell subsets within the spleen, lung and brain on day 60 post‐infection. (h, i) Representative histograms and MFI of OT‐I cells expressing T‐bet in the spleen, lung and brain on day 7 and day 55 post‐infection (FMO control from spleen). Results are combined from two independent experiments. Results are the mean ± SEM of the group (with *n* = 5–6 per group/experiment). (a, c, e) Kruskal–Wallis test with Dunn's post hoc * spleen versus brain; # lung versus brain; $ spleen versus lung *p* < 0·05. (i) One‐way ANOVA with Tukey's post hoc test *p < 0·05

The functionality of the OT‐I cells in the spleen, lung and brain was comparable immediately post‐treatment of ECM on day 7 of PbA‐OVA infection, as measured by GrB and Ki67 expression (Figure 2a,b). The expression of both GrB and Ki67 on OT‐I cells (which were uniformly CD44^hi^) then declined rapidly and at a similar rate in the spleen, lung and brain, with minimal expression at day 60 post‐infection (Figure 2a,b). Thus, memory OT‐I cells did not maintain an effector state or continued proliferation specifically in one tissue site post‐PbA‐OVA infection. Nevertheless, despite equivalent starting phenotypes in the different organs on day 7 of PbA‐OVA infection, the OT‐I cells exhibited increasingly segregated characteristics in the brain, lung and spleen at later time points post‐PbA‐OVA infection (Figure 2c–f). On day 60 post‐infection, OT‐I cells within the brain were primarily CD69^+^CD103^−^KLRG‐1^−^ and CD62L^−^, whereas OT‐I cells within the lung were CD69^–^CD103^–^KLRG‐1^+^ and CD62L^−^ and OT‐I cells within the spleen were predominantly CD69^–^CD103^−^KLRG‐1^−^ and CD62L^+^ (Figure 2c–f). Therefore, memory OT‐I cells that persisted in the brain, lung and spleen post‐PbA‐OVA could be discriminated by the expression of CD69, KRLG‐1 and CD62L, respectively. When we sub‐characterized the memory OT‐I cells into canonical central memory (Tcm: CD44^+^CD62L^+^CD127^+^), effector memory (Tem: CD44^+^CD62L^−^CD127^+^), CD69^+^‐resident memory (CD69^+^CD103^−^Trm: CD44^+^CD62L^−^CD127^+^CD69^+^CD103^−^), CD103^+^‐resident memory (CD103^+^Trm: CD44^+^CD62L^−^CD127^+^CD103^+^CD69^+^), effector (Te: CD44^+^CD62L^−^CD127^−^), terminally differentiated effector (TdTe: CD44^+^CD62L^−^CD127^−^KLRG‐1^+^) effector/memory subsets (gating within spleen as shown in Figure S2), memory OT‐I cells in the brain primarily matched the phenotype of CD69^+^CD103^−^ Trm cells, within the lung the OT‐I cells were predominantly Tem cells and TdTe, and in the spleen the majority of OT‐I cells were Tcm cells (Figure 2g).

Interestingly, the graded expression of T‐bet has been shown to control the diversification of effector and memory T‐cell subsets [43]. Whilst OT‐I cells expressed high levels of T‐bet in the spleen, lung and brain on day 7 of PbA‐OVA infection, with highest expression by cells in the brain, by day 60 post‐infection memory OT‐I cells within the brain expressed significantly lower levels of T‐bet than memory OT‐I cells in the spleen and lung (Figure 2h,i). The distinct specification of the OT‐I cells within the lung and brain (two contrasting non‐lymphoid tissues) post‐PbA‐OVA infection is, therefore, potentially correlated with the level of T‐bet.

Consequently, these data suggest that different signals may impact on memory CD8^+^ T cells within discrete tissues post‐clearance of blood‐stage PbA infection, shaping their diversification and regulating their maintenance.

### The compartmentalization and identity of memory OT‐I cells within the brain following resolution of ECM

**FIGURE 3:**
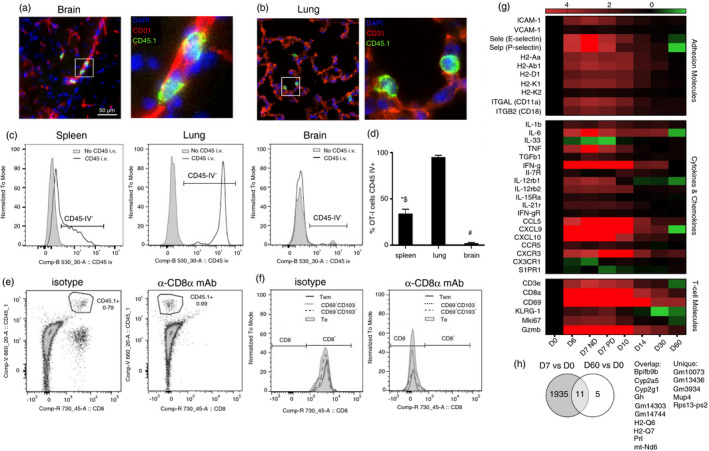
Memory OT‐I cell compartmentalization changes within the brain as neuroinflammation is resolved post‐ECM. 10 000 naïve CD45·1^+^OT‐I cells were adoptively transferred (i.v.) into CD45·2^+^C57BL/6 mice 1 day prior to infection with 10^4^ PbA‐*OVA* pRBCs. The mice were treated (i.p.) with antimalarial drugs (artesunate (30 mg/kg) and chloroquine (30 mg/kg)) when they exhibited signs of ECM on day 6 of infection. (a, b) Immunofluorescence staining showing location of OT‐I cells in (a) brain cortex and (b) lung on day 7 of infection. Mice were administered (i.v.) with 3 μg anti‐CD45‐FITC mAbs for 3 min on day 60 p.i., before tissues were removed (following intracardial whole‐body perfusion with PBS). (c) Representative histograms showing and (d) calculated percentages of CD45·1^+^ OT‐I cells labelled with anti‐CD45‐FITC (CD45 IV^+^) in the spleen, lung and brain (c, compared with control non‐mAb injected mice). Mice were administered (i.p.) with 250 μg anti‐CD8 (clone 53–6·72) or isotype control mAbs for two consecutive days on day 60 p.i., and tissues were removed the following day (day 62) for analysis. (e) Representative dot plots showing capacity to *ex vivo* label brain CD45·1^+^ OT‐I cells from anti‐CD8 mAb and isotype control mAb treated mice with fluorophore‐conjugated anti‐CD8 (53–6·72) mAbs. (f). Representative histograms showing *ex vivo* labelling of gated brain effector and memory CD45·1^+^OT‐I subsets from anti‐CD8 mAb and isotype control mAb treated mice with fluorophore‐conjugated anti‐CD8 (53–6·72) mAbs. Brains were removed on indicated days during PbA infection and after antimalarial drug treatment and RNA‐seq was performed. (g) Heat maps showing the expression kinetics of selected T‐cell relevant genes in the brain during the course of the experiment. (h) Venn diagram contrasting the numbers of genes differentially expressed (< or >1·5‐fold change and q value ≤0·05) in brains on day 7 and day 60 post‐infection, compared with expression in naïve mice. (a, b) Representative images from 3 mice, with 4–8 fields of view captured per animal. (d) Results are combined from two independent experiments. Results are the mean ± SEM of the group (n = 3–4 per group/experiment). One‐way ANOVA with Tukey's post hoc test * spleen versus brain; # lung versus brain; $ spleen versus lung. (g, h) RNA‐seq performed with 4–5 mice per time point

Canonical Trm cells are typically found in the parenchyma of tissues (reviewed [44, 45]). Thus, to examine whether the CD69^+^CD103^−^ memory OT‐I cells within the brain post‐ECM were genuine Trm cells, we qualitatively assessed their compartmentalization. Consistent with previous reports [4, 10, 20, 46], the majority of OT‐I cells within the brain (not including the leptomeninges regions, which were not assessed in this study) on day 7 post‐PbA‐OVA infection were intravascular within small brain capillaries and larger calibre vessels (Figure 3a). Thus, when taken together with previous studies [4, 10, 20, 46], activated antigen‐specific CD8^+^ T cells do not appear to enter the brain parenchyma in high numbers during ECM. Interestingly, despite the subsequent differences in memory phenotype post‐ECM, OT‐I cells within the lung on day 7 post‐PbA‐OVA infection also appeared vascular‐associated, within small alveolar capillaries (Figure 3b).

Unfortunately, as the total numbers of memory OT‐I cells were very low within the brain and lung on day 60 post‐infection, we were unable to accurately analyse their location by histology. Instead, to investigate whether the memory OT‐I cells maintained an intravascular location within the brain and lung on day 60 post‐ECM, we administered anti‐CD45‐FITC i.v. for 3 min, which labels all intravascular (circulating and adherent) cells [35]. Although congestion and extensive occlusion of the cerebrovasculature are hallmarks of ECM (which may reduce the accuracy of this intravascular labelling approach during the established syndrome), vascular obstruction is cleared within the brain by day 14 post‐infection (results not shown). Thus, it is an accurate approach to analyse the compartmentalization of memory OT‐I cells on day 60 post‐infection. Whilst 40% of OT‐I cells in the spleen stained with i.v. administered anti‐CD45‐FITC (were CD45 IV^+^), OT‐I cells within the lung were uniformly CD45 IV^+^. Conversely, OT‐I cells within the brain were almost exclusively CD45 IV^−^ (Figure 3c,d). In the spleen, Tcm cells exhibited a lower trend in CD45 IV staining than Te and TdTe subsets, which likely reflected white pulp and red pulp compartmentalization of the splenic OT‐I populations, whereas in the lung all Tem, Te and TdTe subsets were equally CD45 IV^+^ (Figure S3A,B). Notably, within the brain the Tem, Te, CD69^+^CD103^−^ Trm and CD103^+^ Trm OT‐I cell subsets were equivalently and almost entirely CD45 IV^−^ (Figure S3A,B). Thus, OT‐I cells appear to relocate from a principally luminal to an extravascular location within the brain post‐ECM, and this is independent of their CD69^+^ and CD103^+^ phenotype.

To further address the identity of the extravascular memory CD8^+^ T cell subsets within the brain post‐ECM, we administered anti‐CD8 mAbs (clone 53‐6·72) via i.p. injection to mice for 2 days immediately prior to secondary infection (on day 60 p.i.). It has previously been shown that genuine tissue‐resident/non‐circulating memory CD8^+^ T cells, including those in the CNS, are not accessible to and are not depleted by systemic anti‐CD8 mAb administration [26, 32, 47]. However, all Tem, Te, CD69^+^CD103^−^ Trm and CD69^+^CD103^+^ Trm OT‐I cell subsets in the brain on day 60 p.i. were targeted *in vivo* by the administered anti‐CD8 mAb (as shown by the ability of *in vivo* administered 53–6·72 anti‐CD8mAbs to inhibit the *ex vivo* labelling of CD45·1^+^OT‐I cells with fluorescently conjugated 53.6.72 clone anti‐CD8mAbs; Figure 3e,f). These data, therefore, may suggest that the CD69^+^OT‐I cells in the brain may not be true resident memory T cells. As expected, *in vivo* administered anti‐CD8 mAb also bound to all memory OT‐I cells within the spleen and lung (Figure S3C).

To examine the immunological pathways that may influence the changing intravascular to extravascular positioning of the memory OT‐I cells in the brain post‐ECM, we re‐analysed a whole brain RNA‐seq database covering the time course of recovery from ECM [24]. We biasedly focussed on a number of key genes that have been previously shown to influence memory CD8^+^ T‐cell maintenance and residence (reviewed in [44, 45, 48, 49, 50]). Many genes that influence CD8^+^ T‐cell function and memory maintenance (such as those encoding pro‐inflammatory and homeostatic cytokines and receptors, integrins, MHC and chemokines) were highly upregulated (>1·5 fold change and *q* value ≤0·05) in the brain during ECM (Figure 3g). Thus, the brain appears to have the capacity to shape CD8^+^ T‐cell responses during acute PbA‐OVA infection, potentially reflecting the high level of T‐bet expressed by intracerebral OT‐I cells on day 7 of infection (Figure 2h,i). However, the expression of the immune‐genes, including the expression of adhesion and MHC‐class I molecules, quickly returned to baseline (to that in naïve mice) by day 60 of the experiment (53 days post‐treatment of ECM; Figure 3g). Indeed, the overall brain transcriptome rapidly returned to homeostasis post‐PbA infection, with only 16 genes (which were not enriched within any GO pathways) differentially expressed in the brains on day 60 post‐infection (compared with brains from naïve mice; Figure 3h).

Taken together, these data indicate that brain inflammation is rapidly resolved post‐ECM and that this corresponds with the changed compartmentalization of intracerebral OT‐I cells post‐infection. However, it is unclear whether extravascular OT‐I cells within the brain post‐ECM are genuine Trm cells.

### Memory CD8^+^ T cells are highly pathogenic and are recruited to the brain during secondary PbA‐OVA infection

**FIGURE 4:**
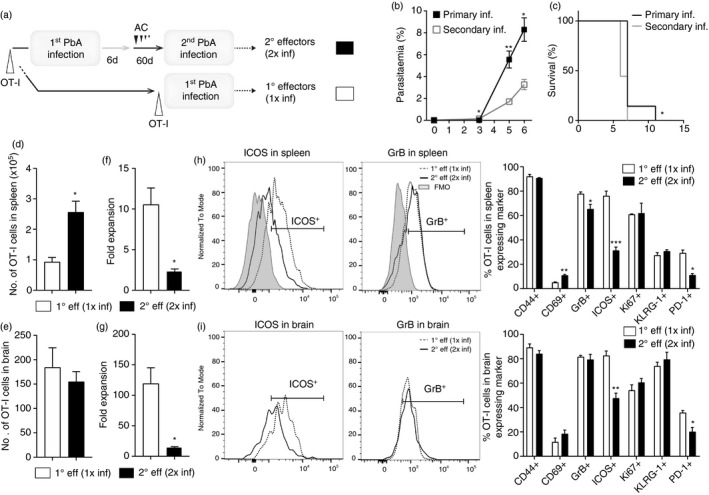
Memory OT‐I cells undergo reactivation and form pro‐inflammatory secondary effector T cells during challenge PbA‐OVA infection. Previously infected and antimalarial drug‐cured CD45·2^+^C57BL/6 mice (which received 10 000 CD45·1^+^OT‐I cells prior to primary infection) were re‐infected (>30 days) with 10^4^ PbA‐OVA pRBCs (secondary infection). Naïve age‐matched CD45·2^+^C57BL/6 mice (which received 10 000 CD45·1^+^OT‐I cells prior to infection) were infected with 10^4^ PbA‐OVA pRBCs (primary infection). (a) Experimental schematic. The course of primary and secondary infection showing (b) peripheral blood parasitaemia, and (c) mortality. The numbers of 1° and 2° effector CD44^+^OT‐I cells within the (d) spleen and (e) brain of primary and secondary infected mice, respectively, when they developed ECM. The fold expansion in 1° and 2° effector CD44^+^ OT‐I cells within the (f) spleen and (g) brain during primary and secondary infection, respectively, calculated relative to precursor OT‐I numbers. The phenotype and activation of 1° and 2° effector CD44^+^ OT‐I cells OT‐I cells within the (h) spleen and (i) brains of primary and secondary infected mice, respectively (FMO from spleen). Results are from one experiment of three independent experiments. Results are the mean ± SEM of the group (n = 4–5 per group). (a, d–g) Unpaired *t* test *p < 0·05, **p < 0·001; (c) Log rank Mantel Cox test *p < 0·05; (h, i) unpaired *t* test or Mann–Whitney tests *p < 0·05, **p < 0·001, ***p < 0·0001

Memory OT‐I cells clearly persisted in/circulated between different tissues (most prominently in the spleen, the dominant secondary lymphoid organ during blood‐stage malaria [40]), post‐PbA‐OVA infection, suggesting the cells may influence the course of challenge PbA‐OVA infections. However, it has previously been proposed that memory CD8^+^ T‐cell reactivation is significantly inhibited during blood‐stage PbA infection due to strong co‐inhibitory receptor‐mediated repression [51]. Consequently, to specifically investigate the reactivation characteristics of the *in vivo* generated memory OT‐I cells during a second challenge blood‐stage PbA‐OVA infection, we re‐infected previously infected mice with PbA‐OVA (re‐infection performed >30 days post‐ACT clearance of primary PbA‐OVA infection parasitaemia: experimental schematic shown in Figure 4a). Re‐infected mice developed ECM with accelerated kinetics during secondary PbA‐OVA infection, compared with mice experiencing a first infection (Figure 4b,c).

Significantly increased numbers of memory‐derived OT‐I cells (2° effector OT‐I cells) were observed within the spleen during a secondary challenge infection than newly activated effector OT‐I cells (1° effector OT‐I cells) within the spleen during a first primary infection (examined at the time of ECM development to ensure analysis of maximal activation of OT‐I cells during each infection, D6 versus D7, respectively; Figure 4d). Moreover, at the time of late‐stage ECM (the peak of T‐cell migration during PbA infection), comparable numbers of 2° effector OT‐I and 1° effector OT‐I cells were observed within the brain during secondary and primary PbA‐OVA infection, respectively (Figure 4e). These data, therefore, show that memory‐derived CD8^+^ T cells are present at high numbers during a secondary PbA‐OVA infection. The precursor numbers of memory OT‐I cells were, however, significantly higher in previously infected mice than the 10 000 naïve OT‐I cells transferred into uninfected mice (for example as shown in Figure 1j). Consequently, the relative expansion of 2° effector OT‐I cells during secondary infection (calculated by dividing the numbers of 2° effector OT‐I cells on day 6 of secondary infection by the number of primary memory OT‐I cells in mice at point of re‐infection) was significantly lower in both the spleen and brain compared with 1° effector OT‐I cells during a first primary infection (calculated by dividing the numbers of 1° effector OT‐I cells on day 7 of infection by number of naïve transferred OT‐I cells at point of primary infection; Figure 4f,g). Thus, memory OT‐I cells may have a proliferative/survival defect upon activation compared with naïve OT‐I cells during PbA‐OVA infection. Despite this, the activation and function of memory‐derived 2° effector OT‐I cells in the spleen and brain during secondary PbA‐OVA infection was comparable to 1° effector OT‐I cells during a primary infection, with memory‐derived 2° effector OT‐I cells and 1° effector OT‐I cells exhibiting very high levels of GrB (Figure 4h,i). Indeed, of the markers examined, 2° effector OT‐I cells and 1° effector OT‐I cells differed only in ICOS expression, which was higher on 1° effector OT‐I cells during primary PbA‐OVA infection (Figure 4h,i). 2° effector OT‐I cells did not have higher expression of co‐inhibitory molecules, such as PD‐1, during a secondary infection compared with 1° effector OT‐I cells during a primary infection, in either the spleen or brain (Figure 4h,i). Therefore, *in vivo* generated antigen‐specific memory CD8^+^ T cells are highly inflammatory, do not exhibit an activation defect and competently accumulate within the brain, during a secondary challenge PbA infection.

### Memory‐derived T cells dominate the parasite‐specific CD8^+^ T‐cell response during secondary PbA‐OVA infection

**FIGURE 5:**
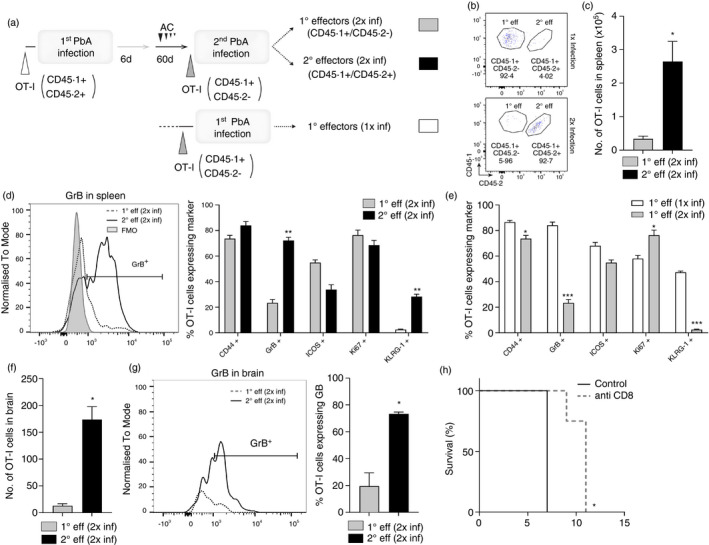
Memory cell‐derived secondary effector T cells dominate the parasite‐specific CD8^+^ T‐cell response during challenge PbA infection. 10 000 naïve CD45·1^+/+^OT‐I cells were adoptively transferred (i.v.) into CD45·2^+^C57BL/6 mice 1 day prior to infection with 10^4^ PbA‐OVA pRBCs. The mice were treated (i.p.) with antimalarial drugs (artesunate (30 mg/kg) and chloroquine (30 mg/kg)) and after >30 days were adoptively transferred 10 000 naïve CD45·1^+/−^OT‐1 cells prior to re‐infection with 10^4^ PbA‐*OVA* pRBCs. 10 000 naïve CD45·1^+/−^OT‐1 cells were transferred to naïve CD45·2^+^C57BL/6 mice prior to primary infection as a control. (a) Experimental schematic. (b) Dot plot representing the gating strategy to identify memory cell‐derived 2° effector CD45·1^+/+^OT‐I cells and 1° effector CD45·1^+/−^OT‐I cells during (top row) primary infection and (bottom row) secondary PbA‐OVA infection. The (c) numbers and (d) phenotype and activation of 2° effector and 1° effector OT‐I cells during secondary challenge infection. (e) The phenotype and activation of 1° effector OT‐I cells during primary and secondary PbA‐OVA infections. The (f) numbers of 2° effector CD45·1^+/+^OT‐I cells and 1° effector CD45·1^+/−^OT‐I cells and (g) the proportions of 2° effector OT‐I cells and 1° effector OT‐I cells producing GrB within the brain during secondary PbA‐OVA infection. (h) The survival of mice undergoing secondary infection treated with anti‐CD8 or control IgG mAbs (250 μg) on days −1, 1, 3, 5 and 7 of secondary infection. Results are from one experiment of three independent experiments. Results are the mean ± SEM of the group (*n* = 3–4 per group). (c–g) Unpaired T test or Mann–Whitney tests *p < 0·05, ** *p* < 0·001, ***p < 0·0001; (h) Log rank Mantel Cox test. *p < 0·05

Memory OT‐I cells clearly reactivated and memory‐derived OT‐I cells accumulated within the brain during a secondary challenge PbA‐OVA infection, suggesting a major role for the memory CD8^+^ T cells in ECM pathology during secondary infection. However, the antigen‐specific CD8^+^ T‐cell response during a second challenge infection can be comprised of both reactivating memory cells (2° effector), as well as newly primed (1° effector) T cells recruited to the response [52]. Therefore, to investigate the relative contribution of 1° effector CD8^+^ T cells to ECM development during secondary PbA‐OVA infection we set up a mixed congenic OT‐I cell adoptive transfer model where we could differentiate memory cell‐derived 2° effector OT‐I cells (CD45·1^+^CD45·2^+^) compared with 1° effector OT‐I cells (CD45·1^+^CD45·2^−^) generated from newly transferred naïve OT‐I cells (experimental schematic in Figure 5a and gating shown in Figure 5b). Using this mixed congenic system, we observed significantly higher numbers of 2° effector OT‐I cells compared with 1° effector OT‐1 cells during secondary PbA‐OVA infection (Figure 5c). The 1° effector OT‐I cells also exhibited significantly reduced function and terminal differentiation (as measured by GrB and KLRG‐1 expression) within the spleen compared with the 2° effector OT‐I cells during secondary infection (Figure 5d), and compared with counterpart 1° effector OT‐I cells generated during a first primary PbA‐OVA infection (Figure 5e). In agreement with this, 1° effector OT‐I cells did not accumulate in high numbers in the brains of mice during secondary challenge infection, and the 1° effector OT‐I cells that did accumulate exhibited significantly reduced effector function, as shown by GrB expression, compared with 2° effector OT‐I cells (Figure 5f,g). Importantly, the expansion and activation of 1° effector OT‐I cells during secondary challenge infection were still restricted even in the absence of pre‐existing memory OT‐I cells, with only a difference in LAG‐3 expression between 1° effector OT‐I cells generated in presence or absence of pre‐existing memory OT‐I cells (Figure S4). Consistent with the observation that systemically administered anti‐CD8 Abs were apparently able to access all memory populations with the spleen, brain and lung post‐infection (Figures 3e,f and S3C), administration of anti‐CD8 mAbs immediately prior to and during challenge infection significantly delayed ECM during secondary PbA‐OVA infection (Figure 5h). This suggests that circulating, and potentially not resident, CD8^+^ T cells promoted ECM during secondary infection.

Collectively, these data show that 2° effector CD8^+^ T cells dominate the antigen‐specific CD8^+^ T‐cell response contributing to ECM development during a secondary challenge PbA infection. Moreover, physiological numbers of endogenous memory CD8^+^ T cells, or the immune environment, preclude naïve CD8^+^ T‐cell recruitment and activation during secondary PbA‐OVA re‐infection.

### Memory CD8^+^ T‐cell responses are suppressed following multiple infections, corresponding with exposure‐acquired resistance against ECM

**FIGURE 6:**
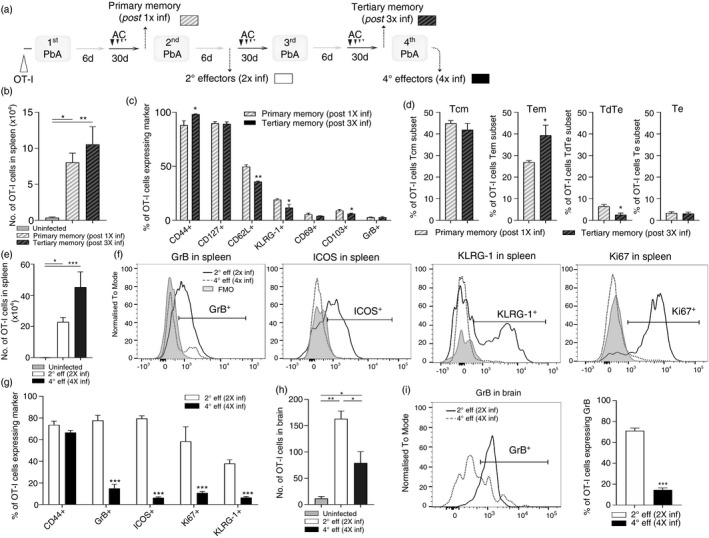
Repeated infection does not impact the maintenance of memory parasite‐specific CD8^+^ T cells, but their reactivation is repressed following multiple infections. CD45·2^+^C57BL/6 mice (adoptively transferred i.v. with 10 000 CD45·1^+^OT‐I cells prior to infection) underwent a single or three rounds of PbA‐OVA infection (10^4^ pRBCs i.v.) – antimalarial drug cure before being re‐infected (>30 days post‐clearance of preceding infection) with PbA‐OVA (second or fourth infection). (a) Experimental schematic. (b) The numbers of primary memory OT‐I cells and tertiary memory OT‐I cells in the spleen of mice post‐first and third infection, respectively (>30 days post‐clearance of preceding infection). (c) The phenotype of primary memory and tertiary memory OT‐I cells in the spleen of mice post‐first and third infection. (d) The proportion of OT‐I cells exhibiting memory T‐cell subset characteristics in the spleen of mice post‐first or third infection. The (e) numbers and (f, g) phenotype of 2° effector OT‐I cells and 4° effector OT‐I cells in spleen of mice during a second and fourth infection (days 6–8 post‐infection), respectively. The (h) numbers of 2° effector OT‐I cells and 4° effector OT‐I cells in the brains of mice during a secondary and fourth infection (days 6–8 post‐infection), respectively, and (i) proportions of 2° effector OT‐I cells and 4° effector OT‐I cells in the brain expressing GrB. (c, g) Results are from one experiment of three independent experiments. Results are the mean ± SEM of the group (*n* = 3–10). (b, d, e, h, i) Results are combined from 2 to 3 separate experiments. Results are the mean ± SEM of the group (*n* = 3–5 per group, experiment). (b, e) Kruskal–Wallis test with Dunn's post hoc. (h) One‐way ANOVA with Tukey's post hoc test. (c, d, g, i) Unpaired *t* test or Mann–Whitney tests. (b–e, g–i) *p < 0·05, **p < 0·001, ***p < 0·0001

Whilst memory‐derived 2° effector CD8^+^ T cells were highly activated during a secondary challenge infection, when mice were highly susceptible to ECM, it has previously been reported that repetitive antigen stimulation can lead to reprogramming of memory CD8^+^ T cells, which substantially influences their reactivation characteristics during recall responses [53, 54]. Of relevance, we have recently shown that three cycles of infection‐drug cure promotes resistance to ECM during a fourth infection, associated with dampened CD8^+^ T‐cell activity [23]. Consequently, we questioned whether the antigen‐specific memory CD8^+^ T‐cell compartment was remodelling following repeated PbA‐OVA infections, and if memory CD8^+^ T cells exhibited repressed reactivation characteristics during a fourth infection, corresponding with resistance to ECM (experimental schematic shown in Figure 6a).

Similar numbers of splenic memory OT‐I cells were maintained (35–60 days post‐drug treatment and parasite clearance) in mice that had experienced one or three prior PbA‐OVA infections (Figure 6b). Moreover, splenic primary memory OT‐I cells (those maintained after one infection) and tertiary memory OT‐I cells (those maintained after three infections) exhibited similar phenotypes, based upon CD44, CD127, CD69, CD103 and GrB expression. Tertiary splenic memory OT‐I cells did, however, express slightly lower levels of CD62L and KLRG‐1 (Figure 6c) than primary memory OT‐I cells. Consequently, repetitive PbA‐OVA challenge infections did not fundamentally alter the magnitude of the splenic memory OT‐I cell pool, but led to a minor skewing of the splenic memory OT‐I cell compartment, with an increase in relative frequencies of Tem cells and a decrease in frequencies of TdTe cells (Figure 6d).

Assessing the reactivation characteristics of the primary and tertiary memory OT‐I cells (into 2° effector and 4° effector cells, respectively), we found that the numbers of 4° effector OT‐I cells trended higher in the spleens of mice during a fourth infection (examined on D8 p.i. when age‐matched primary infected mice developed ECM) compared with 2° effector OT‐I cells in mice during secondary infection (examined on day 6 when the mice developed ECM; Figure 6e). At these corresponding time points during infection, splenic 4° effector OT‐I cells during a fourth infection exhibited significantly reduced activation and function, including lower ICOS and GrB, compared with 2° effector OT‐I cells during secondary infection (Figure 6f,g). Thus, splenic memory OT‐I cell reactivation was dramatically impacted by infection history in repetitively infected mice. In agreement, significantly reduced numbers of 4° effector OT‐I cells accumulated in the brains of mice during a fourth PbA‐OVA infection than 2° effector OT‐I cells in brains of mice during a secondary infection, with intracerebral 4° effector OT‐I cells also showing significantly reduced levels of GrB than intracerebral 2° effector OT‐I cells (Figure 6h,i). As such, these data show that memory OT‐I cell recall effector functions are significantly lowered in repetitively infected mice.

## DISCUSSION

In this study, we have shown that antigen‐specific CD8^+^ T cells are found in various tissue sites, including the brain, post‐drug treatment of ECM. Although activated OT‐I cells exhibited a largely uniform phenotype and function in the spleen, lung and brain during primary PbA‐OVA infection, which likely reflects the systemic nature of infection and the shared splenic ontogeny of the distributed effector T cells [14, 40], the characteristics of the memory OT‐I cells maintained within the discrete tissues diverged increasingly with time post‐primary infection. This suggests that the memory OT‐I cell responses segregating within the brain, lung and spleen may be regulated locally by the individual tissue environments, post‐clearance of PbA‐OVA infection.

The tissue division in memory subsets within the spleen and lung post‐primary PbA infection is consistent with the established model of CD8^+^ T‐cell memory whereby Tem cells persist for prolonged, but not indefinite lengths of time, within non‐lymphoid tissues, providing tissue immunosurveillance, and Tcm circulate between secondary lymphoid tissues, undergoing rapid proliferation upon re‐challenge (reviewed [43, 55]). The observed maintenance of KLRG‐1^+^ OT‐I cells in the lung post‐PbA‐OVA infection was interesting, given the traditional view that KLRG‐1^+^ cells are short‐lived effector T cells [56]. However, long‐lived KLRG‐1^+^IL‐7R^int^ cells have been shown to form after *L*. *monocytogenes* infection [57] and KLRG‐1^+^IL‐7R^+^ memory cells have been shown to differentiate into resident and circulating memory T cells, including Tcm cells [58]. Nevertheless, as the KLRG‐1^+^ cells within the lung post‐PbA were primarily IL‐7R^–^, and as KLRG‐1^+^IL‐7R^–^ cells have high effector function but low proliferative capacity [55], the impact of these KLRG‐1^+^ cells compared with traditional Tem cells for tissue protection/pathology following PbA challenge, their durability, and whether they are influenced by repetitive infection, will require further investigation.

Whilst the phenotype of the memory OT‐I cells within the spleen and lung post‐primary PbA‐OVA infection were largely as expected, the OT‐I cells within the brain exhibited a surprising CD69^+^CD103^−^ phenotype post‐PbA‐OVA infection. In a number of tissue sites CD69^+^CD103^−^ memory CD8^+^ T cells have been classified as Trm cells [44, 45]. Indeed, CD69 is believed to control memory T‐cell retention in tissues through suppressing expression of S1P1, protecting memory T cells from S1P‐dependent egress. [44, 45]. As we, and others, have shown that the vast majority of CD8^+^ T cells recruited to the brain during ECM are intravascular, with large numbers of perivascular/extravascular cells only seen within the leptomeninges [4, 9, 10, 20, 46], we did not expect to identify cells matching the CD69^+^Trm phenotype specifically in the brain post‐ECM. Nevertheless, even though OT‐I cells appeared to change from a primarily intravascular location within the brain during ECM to an extravascular location by day 60 of infection (as defined by lack of labelling with i.v. administered anti‐CD45‐FITC), it is unclear if the CD69^+^ T cells are *bona fide* Trm cells. Neither CD69 nor CD103 expression correlated with lack of CD45‐FITC labelling by OT‐I cells in the brain, with CD69^−^CD103^−^ Tem and Te memory subsets in the brain also excluded from CD45‐FITC i.v. labelling. Moreover, non‐circulating Trm cells within the brain parenchyma have been shown to be resistant to systemic anti‐CD8 mAb administration [26, 32, 47]. We found that the CD69^+^CD103^−^, CD69^+^CD103^+^ and CD69^−^CD103^−^ memory OT‐I cells within the brain on day 60 p.i. were all equally accessible to and were bound by i.p. administered anti‐CD8 mAb, similar to the memory populations within the spleen and lung. Consequently, the accessibility of the memory OT‐I cells within the brain to anti‐CD8 mAb (administered i.p. over 2 days) but not anti‐CD45‐FITC (administered i.v. for only 3 min) may suggest that the memory CD8^+^ T cells within the brain post‐ECM operate within an immunosurveillance programme and transit between the blood and brain extravascular spaces. Thus, further work will be required to fully understand the positioning and activities of the CD69^+^ and CD69^−^ memory CD8^+^ T‐cell subsets in the brain post‐ECM. This will include analysing pro‐inflammatory cytokine production by the intracerebral memory CD8^+^ T‐cell subsets, as Trm cells are characterized by rapid and high levels of IFN‐γ production (reviewed [44, 45]). Notably, although our observation that *in vivo* administered anti‐CD8 mAb bound to but did not deplete memory OT‐I cells was surprising, our results are consistent with a recent report indicating that anti‐CD8mAb clone 53·7.62 binds to and functionally inactivates CD8^+^ T cells (including causing downregulation of the CD8 receptor), but does not promote extensive depletion [59]. Importantly, we and others have used the anti‐CD8 mAb clone 53‐7·62 extensively in murine models of malaria to inhibit CD8^+^ T cell‐mediated pathology [36, 38, 60]

The reasons why memory OT‐I cells compartmentalize differently in the lung (intravascularly) and brain (extravascularly) and adopt different phenotypes post‐ECM is not immediately obvious. During established ECM, our results, along with that of others, suggest that CD8^+^ T cells accumulate within blood vessels and interact with cross‐presenting endothelial cells in equivalent manner in the lung and brain [4, 12, 16, 38]. This would suggest that the OT‐I cells engage with antigen—a key event in shaping intra‐tissue memory immune responses [28, 61]—in a similar way within the lung and brain during PbA‐OVA infection. However, CD69 and KLRG‐1 expression on T cells is also controlled by differing signals, with CD69 expression controlled by TCR signalling, type‐1 interferon, IL‐33 and TNF, whereas IL‐12 and IL‐15 promote KLRG‐1 expression ([56, 57, 58, 61] and reviewed [44, 45]). Relating to this, it was notable that memory OT‐I cells expressed higher levels of T‐bet in the lung than in the brain on day 60 of infection. Inflammation has been shown to promote T‐bet expression in activated CD8^+^ T cells during viral infection, which subsequently controls KLRG‐1 expression [56]. Consequently, this may suggest that the brain and the lung immunological environments differ markedly post‐ECM, sustaining the apparent differences in OT‐I cell compartmentalization and imprinting the observed tissue‐specific cellular phenotypes. Although we did not analyse the lung transcriptome post‐ECM, inflammation was rapidly resolved in the brain post‐ECM.

Our results clearly showed that primary memory OT‐I cell recall was, overall, extremely strong during secondary PbA‐OVA infection. Thus, despite the slightly lower peripheral parasite burden during secondary PbA‐OVA infection, there was apparently sufficient available antigen to allow the memory‐derived 2° effector CD8^+^ T cells to reactivate. As such, our data suggest that primary memory OT‐I cells generated after a single PbA‐OVA infection are not intrinsically defective with limited reactivation potential, although they may exhibit a lower expansion capacity than naïve OT‐I cells (with the caveat that it is difficult to accurately calculate and compare the fold‐change expansion of effector T cells from precursor naïve or memory cells in a tissue during different infections due to the level of effector T‐cell migration that occurs and also the impact of environmental changes within primary and secondary infection). These results, therefore, contrast with a previous report that *in vitro* generated memory OT‐I cells (both Tcm and Tem subsets) are inhibited and are less responsive than naïve OT‐I cells during PbA infection [51]. Indeed, our results suggest the opposite, with *in vivo* generated memory‐derived 2° effector CD8^+^ T cells dominating the pathogenic CD8^+^ T‐cell response during secondary challenge PbA infection with virtually no recruitment of 1° effector CD8^+^ T cells to the immune response. The repression of naïve OT‐I cell activation during challenge PbA‐OVA infection is likely mediated by the pre‐existing memory OVA‐specific CD8^+^ T cells (endogenous and/or transferred OT‐I cells). Indeed, antigen‐specific memory CD8^+^ T cells can restrict the activation of naïve CD8^+^ T cells specific for the equivalent antigen [52, 62]. The relative contribution of the different OT‐I memory cell subsets in the various tissues to the overall 2° effector OT‐I response during challenge PbA infection will require additional investigation.

Whilst primary memory OT‐I cells had very strong reactivation, principally measured by GrB expression, during a second PbA‐OVA infection, the reactivation of tertiary memory OT‐I cells was significantly tempered during a fourth PbA‐OVA infection, when mice are resistant to ECM [23]. Although repetitive stimulation can alter the size of the memory T‐cell compartment in other models [54, 63], the magnitude of the memory OT‐I cell response was equivalent in the spleen after one or three PbA‐OVA infections. Thus, the altered reactivation of tertiary memory OT‐I cells during a fourth infection was not simply a function of increased memory T‐cell numbers. However, in agreement with other studies showing that repetitive antigen stimulation can induce stepwise changes in the memory CD8^+^ T‐cell compartment [52, 53, 63], we observed a slight increase in the proportion of Tem OT‐I cells and a small decrease in the frequencies of TdTe KLRG‐1^+^ OT‐I cells (i.e. long‐lived effector T cells) following three rounds of PbA‐OVA infections. It is clear that different memory CD8^+^ T‐cell populations have differing proliferative, cytokine production and cytolytic capacities upon reactivation [43, 55, 57, 58]. Thus, although more in‐depth investigations such as single‐cell RNA‐seq or proteomics are required to confirm our analyses, repetitive‐stimulation reprogramming or skewing of the maintained splenic memory OT‐I cell compartment may contribute to the repressed reactivation of memory OT‐I cells upon repeated PbA‐OVA infections. Of relevance, repeated *P*. *falciparum* infection also leads to alterations in the phenotype and function of CD8^+^ T cells in children [64], suggesting that such a scenario may be associated with protection against severe malarial disease in humans. Nevertheless, we do not discount the possibility that variations in the inflammatory environment that manifest following repetitive challenge PbA infections, which may include differences in the APC compartment, parasite levels and cytokine production [23, 65, 66], may also contribute to the alterations in memory CD8^+^ T‐cell reactivation in repeatedly PbA‐infected mice. We acknowledge the caveat of normalizing the days of analysis during second (day 6 of infection) and fourth (day 8 of infection) PbA‐OVA infections to the timepoint of ECM development (in the case of four times infected mice, relative to ECM development in control primary PbA‐OVA‐infected mice). This may have introduced temporal differences in, for example, time from initial TCR triggering or dynamics of T‐cell activation into our comparisons of the OT‐I cells in the two infections. Nevertheless, our approach was necessary to analyse the peak splenic activation and subsequent migratory potential of reactivated T cells to the brain during the respective second and fourth PbA‐OVA infections, as maximal T‐cell activation in the PbA model occurs concurrent with ECM development [5, 9, 36, 67].

In summary, our data emphasize the tissue‐specific complexity of the memory CD8^+^ T‐cell response that is generated following blood‐stage PbA infection. This observation has significant implications for considering the relative contribution of specific tissue and memory cell subsets in influencing local and systemic antigen‐specific CD8^+^ T‐cell responses during challenge blood‐stage PbA infections. Moreover, our results suggest that memory CD8^+^ T‐cell reactivation during challenge PbA infections is not stably imprinted, but it is shaped by the number of infection episodes. These observations may have relevance for human malaria, where GrB^+^ CD8^+^ T cells have recently been proposed to play a role in the pathogenesis of severe *P*. *falciparum* malaria [21, 22] and where the nature of memory T‐cell reactivation has been postulated to play a role in susceptibility and resistance against severe malaria disease [68].

## CONFLICT OF INTEREST

The authors declare no competing interests in reference to this manuscript.

## AUTHOR CONTRIBUTIONS

TNS, MJH, JJG, RSD, PS, AC and AVM performed the experiments. TNS, MJH, JJG, PS, LZ, KNC analysed the data. TNS and KNC designed the study. TNS and KNC wrote and edited the manuscript.

## Supporting information

Fig S1

Fig S2

Fig S3

Fig S4

Supplementary Material
