# Supplementary figures and images for: Memory CD8+ T cells exhibit tissue imprinting and non‐stable exposure‐dependent reactivation characteristics following blood‐stage Plasmodium berghei ANKA infections
Source: Immunology. 2021 Aug 27;164(4):737–53. doi: 10.1111/imm.13405 (PMC8561116; doi:10.1111/imm.13405)

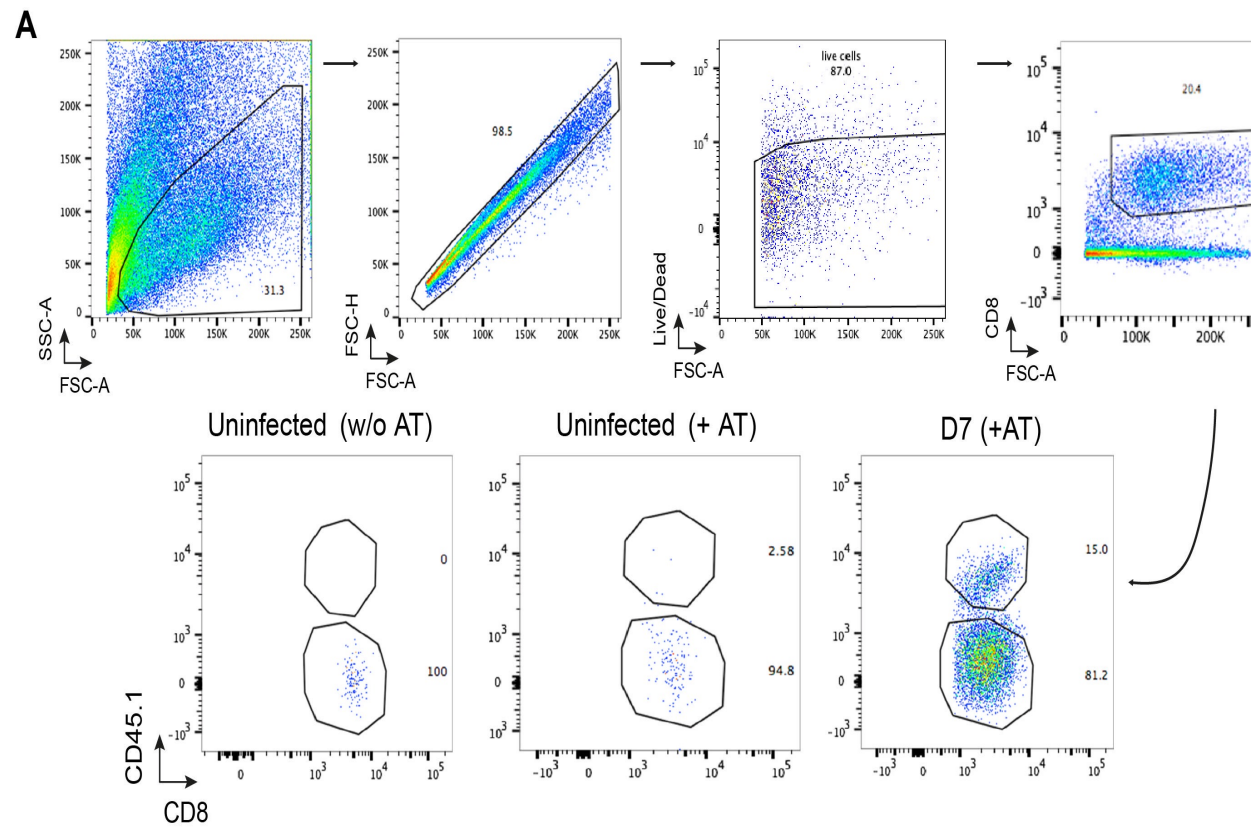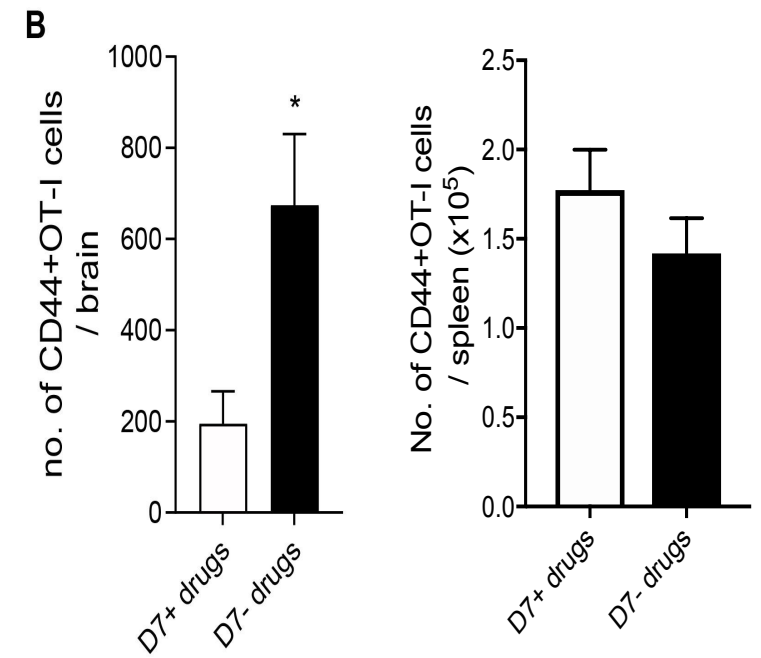

Supplement: Supplementary file 1 — Fig S1 [file IMM-164-737-s003.pdf]

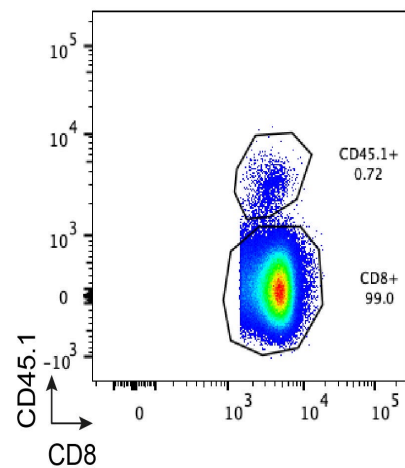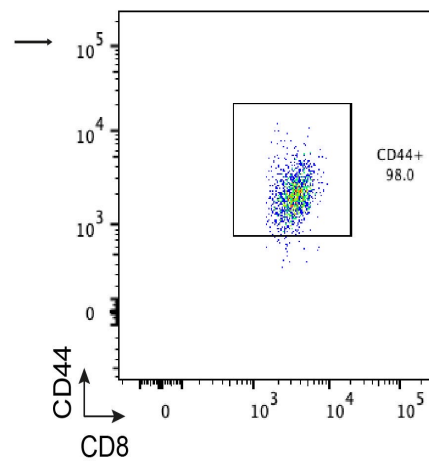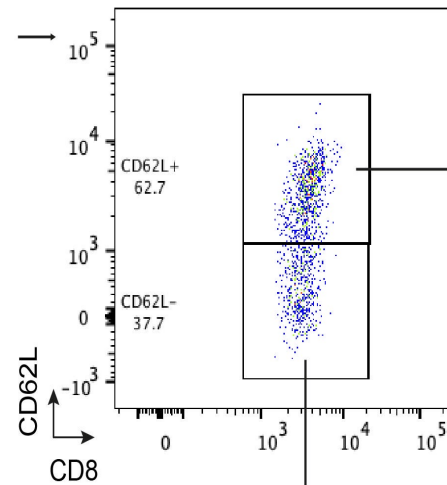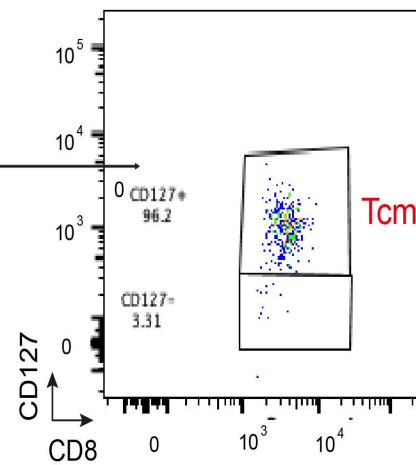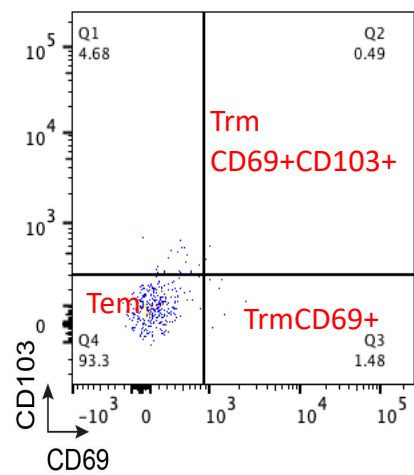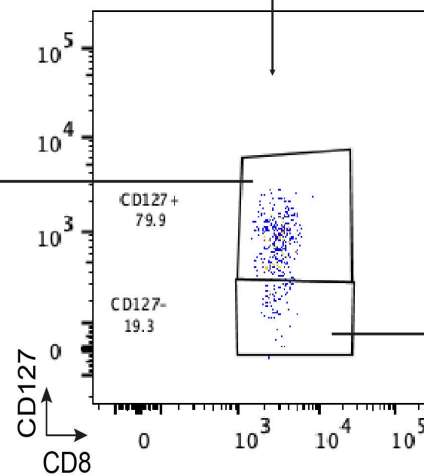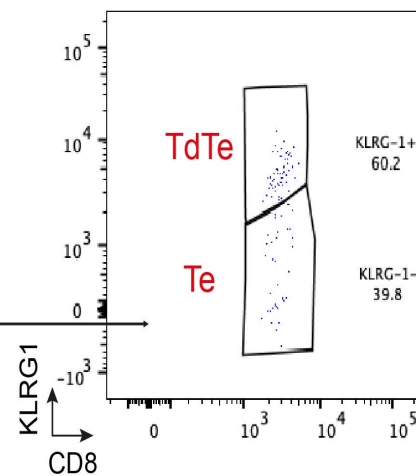

Supplement: Supplementary file 2 — Fig S2 [file IMM-164-737-s002.pdf]

A

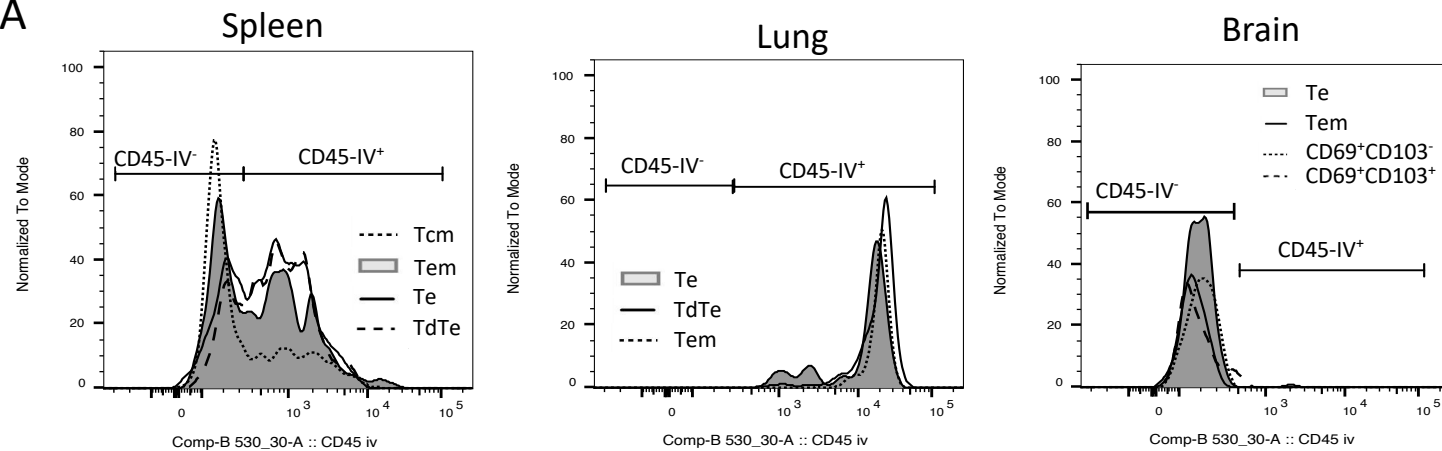

B

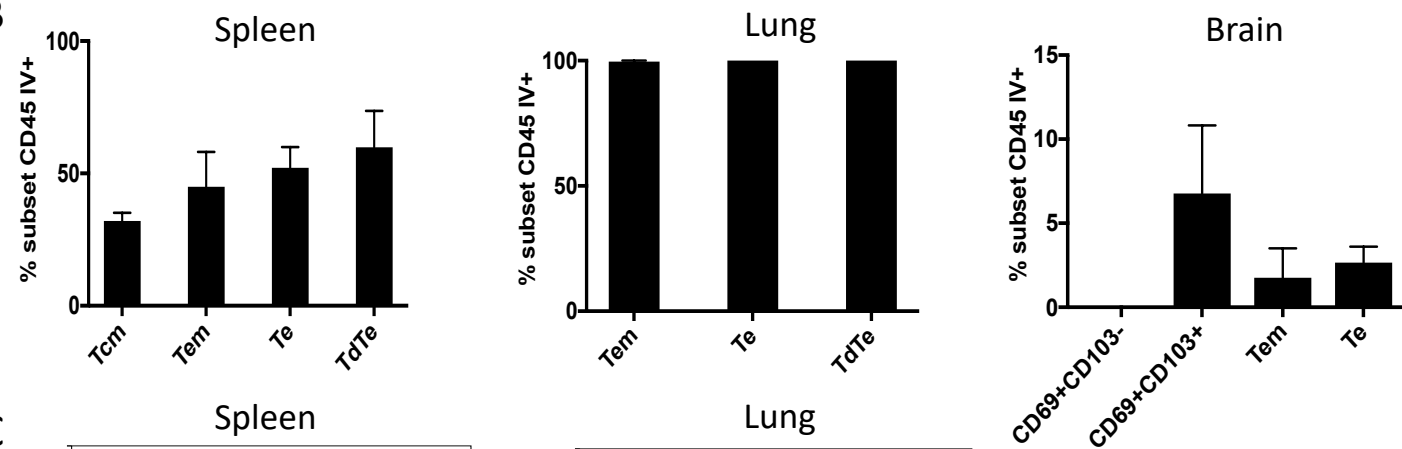

C

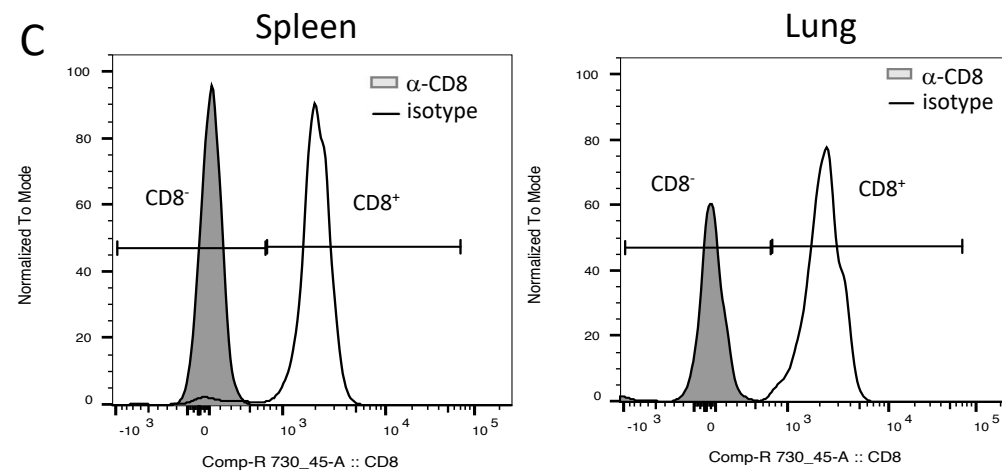

Supplement: Supplementary file 3 — Fig S3 [file IMM-164-737-s004.pdf]

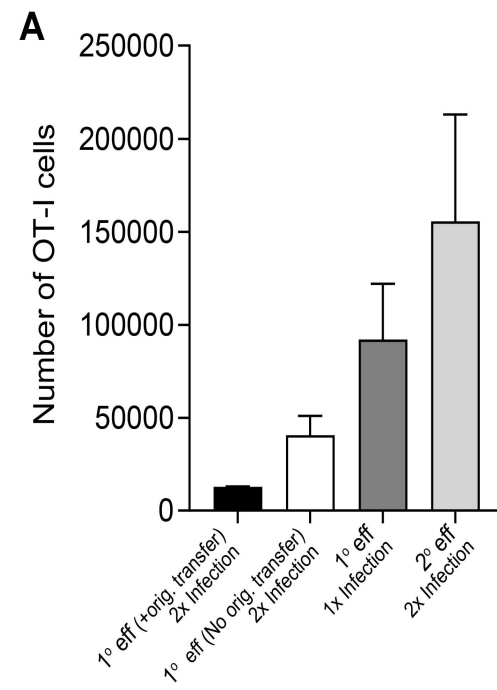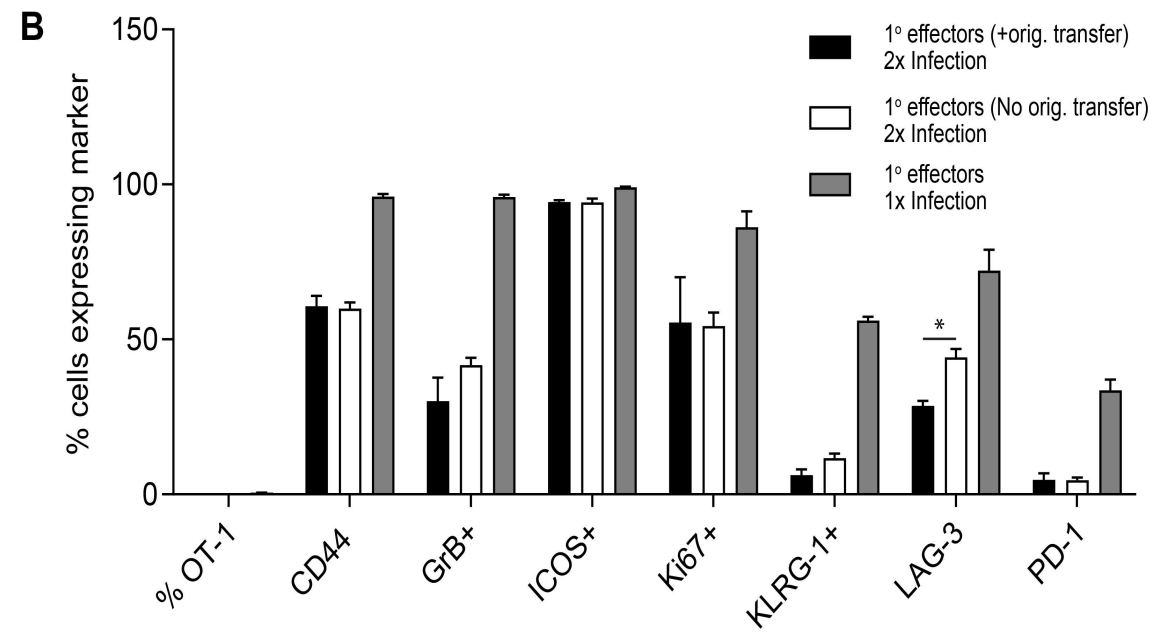

Supplement: Supplementary file 4 — Fig S4 [file IMM-164-737-s005.pdf]
