# Supplementary material for: Memory CD8+ T cells exhibit tissue imprinting and non‐stable exposure‐dependent reactivation characteristics following blood‐stage Plasmodium berghei ANKA infections
Source: Immunology. 2021 Aug 27;164(4):737–53. doi: 10.1111/imm.13405 (PMC8561116; doi:10.1111/imm.13405)
Supplement: Supplementary file 5 — Supplementary Material [file IMM-164-737-s001.docx]

**Supplementary Figures**

**Supplementary Figure 1: Identification of OT-I cells within the brain of mice following PbA-OVA infection.**

10,000 naïve CD45.1^+^OT-I cells were adoptively transferred (i.v.) into CD45.2^+^C57BL/6 mice one day prior to infection with 10^4^ PbA*-OVA* pRBCs. The mice were treated (i.p.) with anti-malarial drugs (artesunate (30mg/kg) and chloroquine (30mg/kg)) when they exhibited signs of ECM. (**A**) Gating strategy to identify CD45.1^+^ OT-I cells in the brain. (**B**) The numbers of effector CD44^+^ OT-I cells in the brain and spleen of mice on day 7 with /without anti-malarial drug treatment. (**B**) Results are from one experiment of three independent experiments. Results are the mean + /- SEM (with n=5-6 per group). Mann Whitney Test * p<0.05

**Supplementary Figure 2: Gating strategy to identify memory OT-I cell subsets**

10,000 naïve CD45.1^+^OT-I cells were adoptively transferred (i.v.) into CD45.2^+^C57BL/6 mice one day prior to infection with 10^4^ PbA*-OVA* pRBCs. The mice were treated (i.p.) with anti-malarial drugs (artesunate (30mg/kg) and chloroquine (30mg/kg)) when they exhibited signs of ECM. Representative gating (from spleen on day 60 post-infection) defining identification of central memory (Tcm: CD44^+^CD62L^+^CD127^+^), effector memory (Tem: CD44^+^CD62L^-^CD127^+^), CD69^+^CD103^-^ resident memory (CD69^+^Trm: CD44^+^CD62L^-^CD127^+^CD69^+^), CD103^+^ resident memory (CD103^+^Trm: CD44^+^CD62L^-^CD127^+^CD103^+^CD69^+^), effector (Te: CD44^+^CD62L^-^CD127^-^), terminally differentiated effector (TdTe: CD44^+^CD62L^-^CD127^-^KLRG-1^+^) effector / memory subsets.

**Supplementary Figure 3: The compartmentalisation of OT-I cells in the spleen, lung and brain on day 60 post-infection**

10,000 naïve CD45.1^+^OT-I cells were adoptively transferred (i.v.) into CD45.2^+^C57BL/6 mice one day prior to infection with 10^4^ PbA*-OVA* pRBCs. The mice were treated (i.p.) with anti-malarial drugs (artesunate (30mg/kg) and chloroquine (30mg/kg)) when they exhibited signs of ECM on day 6 of infection. (**A, B**) Mice were administered (i.v.) on day 60 p.i. with 3μg anti-CD45-FITC mAbs for 3 minutes before tissues were removed (following intracardial whole body perfusion with PBS). (**A**) Representative histograms showing and (**B**) calculated percentages of gated effector and memory OT-I cell subsets labelled with anti-CD45-FITC (CD45-IV^+^) in the spleen, lung and brain. (**C**) Mice were administered (i.p.) with 250μg anti-CD8 (clone 53-6.72) or isotype control mAbs for two consecutive days on day 60 p.i. (**C**) Representative histograms showing *ex vivo* labelling of spleen and lung CD45.1^+^ OT-I cells from anti-CD8 mAb and isotype control mAb treated mice with fluorophore-conjugated anti-CD8 (53-6.72) mAbs. (**B**) Results are from one experiment of two independent experiments. Results are the mean + /- SEM of the group (with n= 3-4 per group).

**Supplementary Figure 4: 1^o^ effector OT-I cell responses are defective during secondary PbA-OVA infection even in absence of pre-existing memory OT-I cells**

CD45.2^+^C57BL/6 mice received or did not receive 10,000 naïve CD45.1^+/+^OT-I cells (i.v.) one day prior to infection with 10^4^ PbA*-*OVA pRBCs. The mice were treated (i.p.) with anti-malarial drugs (artesunate (30mg/kg) and chloroquine (30mg/kg)) and after >30 days were adoptively transferred 10,000 naïve CD45.1^+/-^OT-1 cells prior to re-infection with 10^4^ PbA*-OVA* pRBCs. 10,000 naïve CD45.1^+/-^OT-1 cells were transferred to naïve CD45.2^+^C57BL/6 mice prior to primary PbA-OVA infection as a control. (**A**) The numbers of 1^o^ effector CD44^+^ OT-I cells in secondary infected mice that had received or not received an adoptive transfer of OT-I cells prior to primary infection (compared with the numbers of 1^o^ effector and 2^o^ effector cells in primary and secondary PbA-OVA infected mice, respectively). (**B**) The activation state of splenic 1^o^ effector OT-I cells in groups of primary and secondary PbA-OVA infected mice specified in (**A**). Results are from one experiment of two independent experiments (with n=5-6 per group).
